# Transferrin receptor 1 deficiency in smooth muscle cells attenuates vascular remodeling in hypertensive mice

**DOI:** 10.1038/s41440-025-02254-4

**Published:** 2025-06-09

**Authors:** Yoshiro Naito, Hisashi Sawada, Tetsuo Horimatsu, Masaki Ohmuraya, Masanori Asakura, Masaharu Ishihara

**Affiliations:** 1 Department of Cardiovascular and Renal Medicine, School of Medicine, Hyogo Medical University, Nishinomiya, Japan; 2 Saha Cardiovascular Research Center, College of Medicine, University of Kentucky, Lexington, KY USA; 3 Saha Aortic Center, College of Medicine, University of Kentucky, Lexington, KY USA; 4 Department of Physiology, College of Medicine, University of Kentucky, Lexington, KY USA; 5 Department of Genetics, School of Medicine, Hyogo Medical University, Nishinomiya, Japan

**Keywords:** Transferrin receptor 1, Vascular remodeling, Smooth muscle cell

## Abstract

Transferrin receptor 1 (TfR1), a crucial cellular iron receptor, has a variety of biological functions. We have previously reported that TfR1 abundance increases in the aortic media of hypertensive rat models. However, its role in vascular diseases remains unknown. In the present study, we generated smooth muscle cell (SMC)-specific TfR1 deficient mice and examined the impact of its deletion in mouse models of hypertension induced by angiotensin II or deoxycorticosterone acetate/salt administration. SMC-specific TfR1 deletion attenuated medial thickening and elastin fragmentation in both mouse models of hypertension. These results indicate that TfR1 in SMCs exerts a role in vascular remodeling in hypertension.

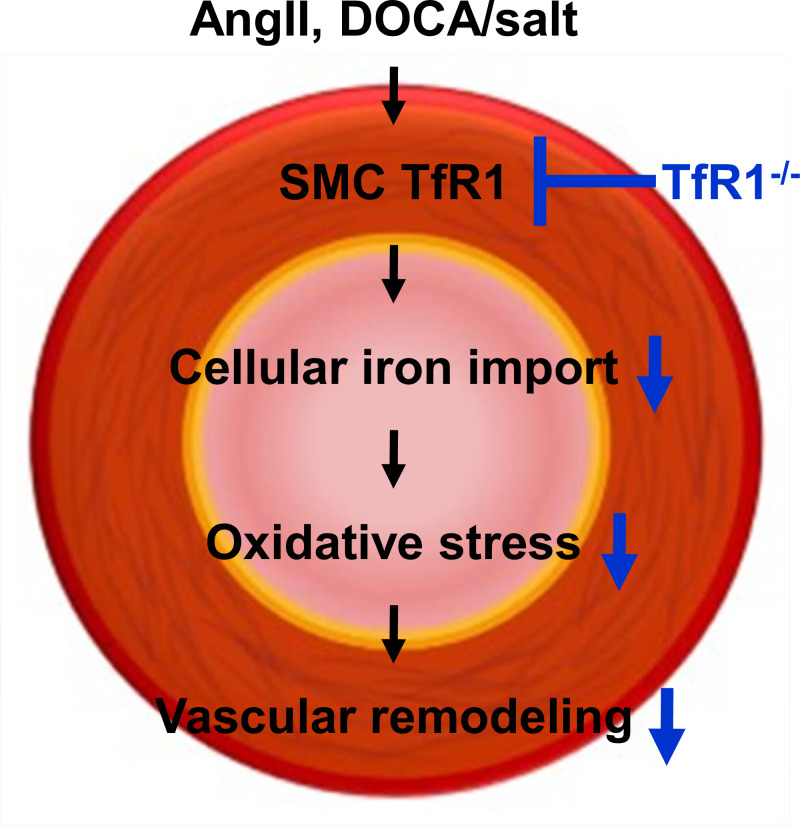

## Introduction

Transferrin receptor 1 (TfR1) is a cellular iron receptor that is essential for hematopoiesis in erythroid cells [1]. Recent studies have shown a variety of its biological functions in non-hematopoietic cells [2–4]. In the heart, TfR1 contributes to iron uptake by cardiomyocytes. TfR1 deficiency in cardiomyocytes leads to dilated cardiomyopathy in mice [4]. In vascular diseases, we have previously reported that TfR1 abundance increases in the aortic media of hypertensive rat models [5, 6]. However, its biological role in vascular diseases remains unknown.

Vascular remodeling, such as medial thickening and extracellular matrix degradation, is a prominent feature of vascular diseases [7]. Smooth muscle cells (SMCs) are the major cellular component of the aortic media and exert a critical role in medial thickening. Considering our previous results that TfR1 abundance increases in the aortic media of hypertensive rat models, in the present study, we hypothesized that TfR1 in SMCs is associated with the pathogenesis of vascular remodeling in hypertension. To test this hypothesis, we generated SMC-specific TfR1 deficient mice and investigated the impact of its deletion in mouse models of hypertension.

## Methods

### Animals

All experimental procedures were approved by the Animal Research Committee of Hyogo Medical University (Protocol #19-039, #22-010AG, #25-047AG). *Myosin heavy chain (Myh)11-CreER*^*T2*^ + /0 mice (#019079, The Jackson Laboratory) were crossbred to *Tfr1* floxed mice (#028363, The Jackson Laboratory) to generate *Tfr1* floxed *Myh11-CreER*^*T2*^ + /0 mice. SMC-specific TfR1 deficient (SMC-TfR1–/–) mice were generated through intraperitoneal tamoxifen injection (75 mg/kg/day for 5 days, Sigma-Aldrich) in *Tfr1* floxed *Myh11-CreER*^*T2*^ + /0 mice. *Tfr1* floxed *Myh11-CreER*^*T2*^ + /0 mice without tamoxifen injection were used as controls. As the *Myh11-CreER*^*T2*^ transgene is located on the Y chromosome, the present study used only male mice.

### Mouse models of hypertension

Mice were infused with angiotensin II (AngII; 1000 ng/kg/min, Peptide Institute) subcutaneously for 4 weeks via an osmotic mini-pump. In addition, as another model of hypertension, deoxycorticosterone acetate (DOCA)/salt hypertension was induced in mice. DOCA pellets (50 mg, Innovative Research of America) were implanted subcutaneously for 3 weeks in mice, after uninephrectomy, and NaCl (0.9%, wt/vol) was added to their drinking water.

### Assessments of systolic blood pressure

Systolic blood pressure (SBP) was measured by a non-invasive tail-cuff system (MK-2000, Muromachi Kikai) [8].

### Genomic DNA polymerase chain reaction

Following tamoxifen-induced recombination, thoracic aortic tissue was dissected from mice. A small piece of the whole aorta was processed for genomic DNA extraction. Polymerase chain reaction was performed using two primer pairs: (1) the proximal *flox* sequence in intron 2 (5ʹCAGTAATCCCAGAGGAATCATTAG3ʹ and 5ʹCTAAACCGGGTGTATGACAATG3ʹ, 650 bp), (2) *Tfr1* delta *flox* fragment (5ʹAGTCAGGGCTAGTCTGAGCA3ʹ and 5ʹAGTACTTGGGAGGCAAAGGC3ʹ, 507 bp).

### Western blot analysis

The protein homogenate was separated by SDS-PAGE and transferred onto polyvinylidene fluoride membranes. Anti-TfR1 antibody (Invitrogen; dilution 1:1000), anti-ferritin antibody (abcam; dilution 1:1000), anti-divalent metal transporter-1 (DMT1) antibody (abcam; dilution 1:1000), and anti-glyceraldehyde-3-phosphate dehydrogenase (GAPDH) antibody (Cell Signaling Technology; dilution 1:1000) were used as the primary antibodies. The protein bands were illuminated by chemiluminescence kits (Thermo Fisher Scientific).

### Histological analysis

The aorta tissues were embedded in Tissue-Tek OCT compound (Sakura Finetechnical, Co) and cut into 10-μm-thick sections. Masson’s trichrome staining and Elastica-van Gieson staining were performed using serial aortic sections. Medial thickness and elastin fiber breaks were measured in 3 serial ×40 magnification images by ImageJ software. Immunohistochemical staining was performed with anti-TfR1 antibody (abcam; 2 µg/mL) and anti-4-hydroxynonenal (4HNE) antibody (abcam; 3.1 µg/mL), and visualized using an avidin-biotin-peroxidase conjugate and 3-amino-9-ethylcarbazole substrate. Every section was counterstained with hematoxylin.

### Statistical analysis

Data were expressed as the means ± SEM. All numerical data passed the Shapiro-Wilk normality test. *P* values were determined by two-way ANOVA followed by Holm-Sidak test using GraphPad Prism 9. Values of *P* < 0.05 were considered statistically significant.

## Results

**Fig. 1:**
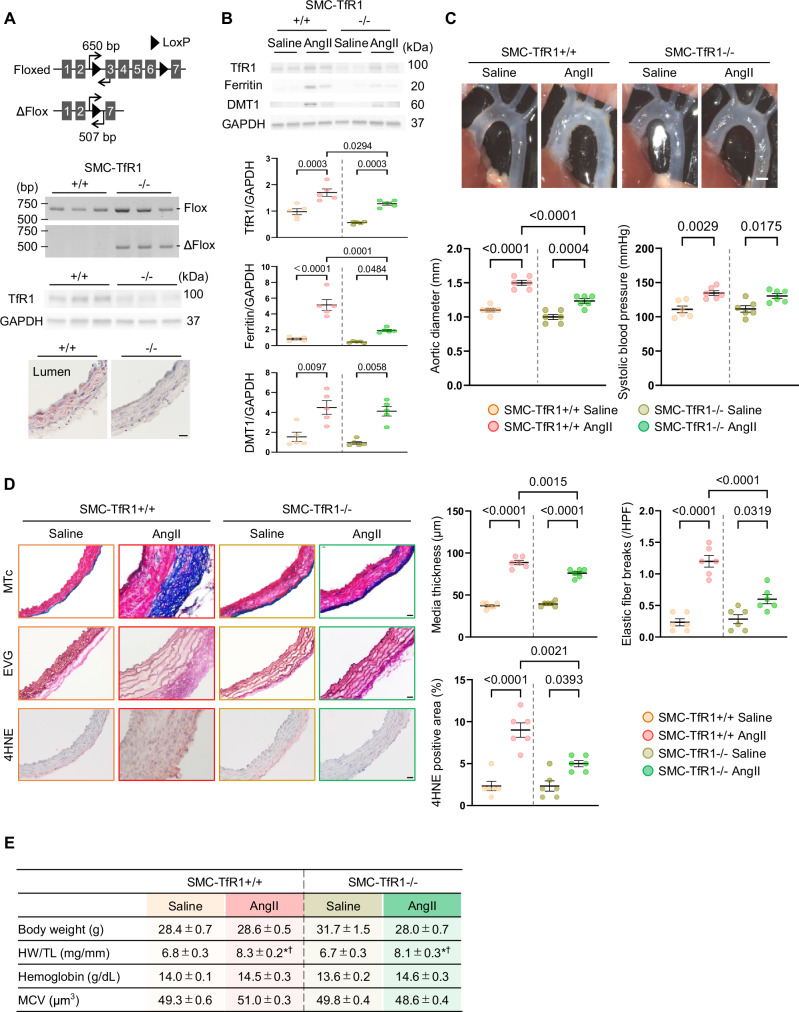
TfR1 Deficiency in Smooth Muscle Cells Attenuates Vascular Remodeling in Mice after AngII Administration. **A** Schematic illustration of Floxed and null (ΔFlox) alleles for TfR1 deletion in *Tfr1* floxed *Myh11-CreER*^*T2*^ + /0 mice. Polymerase chain reaction, Western blot analysis, and representative images of immunostaining for TfR1 in the aorta of SMC-TfR1 + /+ and –/– mice (scale bar: 20 µm). **B** Western blot analysis for aortic TfR1, ferritin, and DMT1, **C** representative in situ images, external diameters of the thoracic aorta, and systolic blood pressure in SMC-TfR1 + /+ and –/– mice with either saline or AngII infusion (scale bar: 1 mm). *n* = 5–6/group. **D** Representative images of Masson’s trichrome (MTc), Elastica-van Gieson (EVG), and 4-hydroxynonenal (4HNE) staining in the aortic sections (scale bar: 20 µm), media thickness, elastin fiber breaks, and quantitative analysis of 4HNE-positive area in SMC-TfR1 + /+ and –/– mice with either saline or AngII infusion. *n* = 6/group. **E** Body weight, heart weight (HW)/tibia length (TL), hemoglobin, and mean corpuscular volume (MCV) in SMC-TfR1 + /+ and –/– mice with either saline or AngII infusion. ^*^p < 0.05 vs SMC-TfR1 + /+ mice with saline infusion. ^†^*p* < 0.05 vs SMC-TfR1–/– mice with saline infusion. *n* = 5–6 per group

We firstly validated recombination of Tfr1 DNA by the detection of Tfr1 delta flox fragment (Fig. 1A). SMC-TfR1–/– mice exhibited bands for both the *flox* sequence and delta *flox* fragment. Aortic TfR1 protein abundance was significantly decreased in SMC-TfR1-/- mice in Western blot analysis, and TfR1 positive cells were not observed in the aortic media in immunostaining (Fig. 1A). These data were solid evidence for the successful deletion of TfR1 in SMCs of the model mice.

Using these mice, we determined the impact of TfR1 deletion in SMCs on vascular remodeling in hypertension induced by AngII. Ascending aortas were harvested 4 weeks after either saline or AngII infusion. Aortic TfR1 protein abundance was increased by AngII infusion in SMC-TfR1 + /+ mice, whereas its elevation was suppressed in SMC-TfR1–/– mice (Fig. 1B). Aortic ferritin protein abundance also increased in mice after AngII infusion, and this elevation was attenuated in SMC-TfR1–/– mice (Fig. 1B). Aortic DMT1, endosomal iron transporter, protein abundance also increased in mice after AngII infusion; however, this elevation was comparable in both genotypes (Fig. 1B). AngII infusion increased the external diameters of the ascending aorta of SMC-TfR1 + /+ mice, whereas it was less severe in SMC-TfR1-/- mice (Fig. 1C). SBP was increased comparably in both genotypes, demonstrating that the attenuation was independent of blood pressure. In addition, significant inhibition of AngII-induced medial thickening, elastin fragmentation, and an oxidative stress marker, 4HNE-positive area was observed in SMC-TfR1-/- mice (Fig. 1D). Regarding to physiological and hematological parameters, body weight did not differ among the experimental groups. AngII infusion increased heart weight to tibia length ratio similarly in both genotypes. Hemoglobin content and mean corpuscular volume were not statistically significant among the experimental groups (Fig. 1E). These data support the notion that TfR1 in SMCs exerts a role in vascular remodeling in hypertension.

**Fig. 2:**
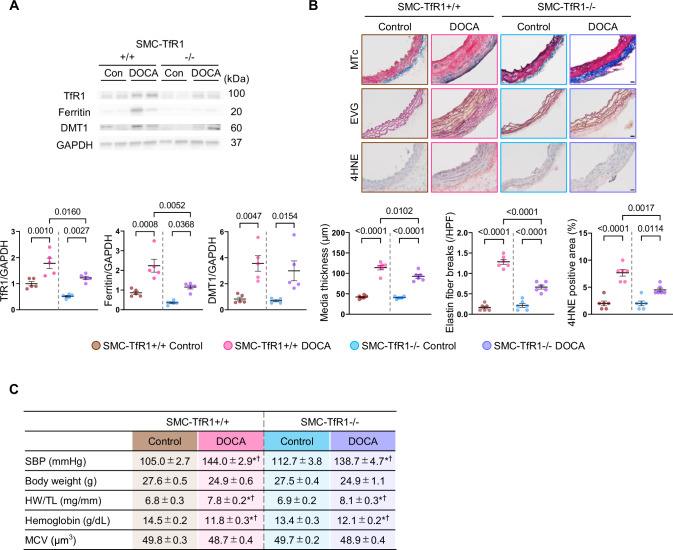
TfR1 Deficiency in Smooth Muscle Cells Attenuates Vascular Remodeling in Mice after DOCA/salt Administration. **A** Western blot analysis for aortic TfR1, ferritin, and DMT1 in SMC-TfR1 + /+ and -/- mice with either DOCA/salt or sham/water administration. *n* = 5/group. **B** Representative images of Masson’s trichrome (MTc), Elastica-van Gieson (EVG), and 4-hydroxynonenal (4HNE) staining in the aortic sections (scale bar: 20 µm), media thickness, elastin fiber breaks, and quantitative analysis of 4HNE-positive area in SMC-TfR1 + /+ and –/– mice with either DOCA/salt or sham/water administration. *n* = 6/group. **C** Systolic blood pressure (SBP), body weight, heart weight (HW)/tibia length (TL), hemoglobin, and mean corpuscular volume (MCV) in SMC-TfR1 + /+ and –/– mice with either DOCA/salt or sham/water administration. ^*^p < 0.05 vs SMC-TfR1 + /+ mice with sham/water administration. ^†^*p* < 0.05 vs SMC-TfR1–/– mice with sham/water administration. *n* = 5–6 per group

Since previous studies have reported that AngII has direct effects on vascular remodeling independent of blood pressure increase [9], the impact of TfR1 deletion in SMCs was further determined in another model of hypertension, DOCA/salt hypertensive mice. Consistent with the results in AngII infused mice (Fig. 1B, D), aortic TfR1 and ferritin protein abundance was increased by DOCA/salt administration regardless of genotype; however, it was modest in mice with TfR1 deletion in SMCs (Fig. 2A). Aortic DMT1 protein abundance was similarly increased by DOCA/salt administration in both genotypes (Fig. 2A). Medial thickening, elastin fragmentation, and 4HNE-positive area were attenuated significantly in SMC-TfR1–/– mice (Fig. 2B). As shown in Figure 2C, SBP was not different between genotypes in response to DOCA/salt administration. Body weight was comparable among the experimental groups. Heart weight to tibia length ratio was increased similarly in both genotypes after DOCA/salt administration. Hemoglobin content was decreased by uninephrectomy regardless of genotype, while mean corpuscular volume was not different among the groups.

## Discussion

The present study demonstrated that SMC-specific TfR1 deletion attenuates medial thickening and elastic fiber fragmentation in mouse models of hypertension.

Of note, the protective effects of TfR1 deletion in SMCs were observed in not only AngII but also DOCA/salt administered mice, suggesting that TfR1 contributes to vascular remodeling in hypertension independent of AngII mediated mechanisms. Oxidative stress is associated with both AngII and DOCA/salt induced hypertension [10]. There is compelling evidence that iron is also associated with oxidative stress, important drivers for vascular remodeling. Since aortic 4HNE-positive area was attenuated by TfR1 deletion, along with reduced aortic TfR1 and ferritin protein abundance, the inhibition of vascular remodeling in SMC-TfR1-/- mice may be associated with oxidative stress. In addition, iron is a key cofactor for enzymes involved in cell proliferation. Medial thickening is attributed to cell proliferation centered on SMCs. As aortic ferritin protein abundance was attenuated by TfR1 deletion, TfR1 deletion in SMCs may suppress cell proliferation by inhibiting iron uptake into cells, thereby attenuating medial thickening. In these regards, it is fascinating to elucidate the mechanism by which TfR1 deficiency in SMCs suppresses vascular remodeling in hypertensive mice.

As TfR1 is essential for hematopoiesis in erythroid cells, global TfR1 deficient mice exhibit abnormalities in erythropoiesis [1]. We have previously reported that TfR1 heterozygous deficient mice showed decreased hypoxia-induced pulmonary vascular remodeling compared to wild-type controls [11]. In the previous study, TfR1 heterozygous deficient mice did not exhibit anemia, but had a smaller mean corpuscular volume compared to controls. Thus, TfR1 heterozygous deletion in erythroid cells may contribute to the attenuation of hypoxia-induced pulmonary vascular remodeling in these mice. To rule out the impact of TfR1 in erythroid cells, the present study generated SMC-specific TfR1 deficient mice. SMC-specific TfR1 deletion reduced vascular remodeling in hypertension without affecting erythropoiesis. These results indicate that TfR1 in SMCs plays a role in vascular remodeling in hypertension independent of erythropoiesis.

Although several studies have reported various mechanisms underlying vascular remodeling in hypertension [7, 12], the mechanisms are complex and not fully defined yet. Our results may provide new insights into understanding the mechanisms of vascular remodeling in hypertension.

In conclusion, we demonstrate direct in vivo evidence that TfR1 in SMCs exerts a role in vascular remodeling in hypertension.
